# Survey of domestic cattle for anti-*Leishmania *antibodies and *Leishmania *DNA in a visceral leishmaniasis endemic area of Bangladesh

**DOI:** 10.1186/1746-6148-7-27

**Published:** 2011-06-08

**Authors:** Mohammad Shafiul Alam, Debashis Ghosh, Md Gulam Musawwir Khan, Mohammad Faizul Islam, Dinesh Mondal, Makoto Itoh, Md Nurul Islam, Rashidul Haque

**Affiliations:** 1 ICDDR, B, 68 Shaheed Tajuddin Ahmed Sarani, Mohakhali, Dhaka 1212, Bangladesh; 2 Department of Parasitology, Aichi Medical University School of Medicine, Nagakute, Aichi, Japan; 3 Upazila Livestock Officer, Trishal, Mymensingh, Bangladesh

## Abstract

**Background:**

Visceral leishmaniasis (VL), caused by an intracellular parasite *Leishmania donovani *in the Indian subcontinent, is considered to be anthroponotic. The role of domestic animals in its transmission is still unclear. Although cattle are the preferred blood host for *Phlebotomus argentipes*, the sandfly vector of VL in the Indian subcontinent, very little information is available for their role in the disease transmission. In this study, we examined domestic cattle for serological and molecular evidence of *Leishmania *infection in a VL-endemic area in Bangladesh. Blood samples from 138 domestic cattle were collected from houses with active or recently-treated VL and post-kala-azar dermal leishmaniasis patients. The presence of anti-leishmanial antibodies in serum was investigated using enzyme-linked immunosorbent assay (ELISA) and then with direct agglutination tests (DAT). Nested PCR (Ln PCR) was performed to amplify the *ssu-rRNA *gene using the DNA extracted from Buffy coat. Recently-developed molecular assay loop-mediated isothermal amplification (LAMP) was also performed for further sensitive detection of parasite DNA.

**Results:**

In this study, 9.4% (n = 13) of the cattle were found to be positive by ELISA. Of the 13 ELISA-positive cattle, only four (30.8%) were positive in DAT. Parasite DNA was not detected in either of the molecular assays (Ln PCR and LAMP).

**Conclusions:**

The study confirmed the presence of antibodies against *Leishmania *parasite in cattle. However, the absence of *Leishmania *DNA in the cattle indicates clearly that the cattle do not play a role as reservoir host. Similar study needs to be undertaken in the Indian subcontinent to determine the role of other domestic animals on which sandflies feed.

## Background

Eighty-eight countries of the world are endemic with either of the two major forms of leishmaniasis: cutaneous leishmaniasis (CL), a disfiguring and stigmatizing disease, and visceral leishmaniasis (VL) or kala-azar, which is fatal if remains untreated [1]. One hundred fifty million people are living with the risk of VL in the Indian subcontinent (India, Nepal, and Bangladesh) [2]. VL leads to a loss of about 400,000 disability-adjusted life-years (DALYs) every year in this region [3]. VL is believed to be anthroponotic in the subcontinent. Results of several studies have shown that *Phlebotomus argentipes*, the only known vector for *Leishmania donovani *in the Indian subcontinent, prefer to feed on both bovine and human blood [4-8]. Being a preferable host for *P. argentipes*, cattle was shown to play an undecided role in several epidemiological studies in the Indian subcontinent [9]. For example, Ownership of cattle in Nepal and its density in Bangladesh were found to be protective [10,11]. Whereas, increased risk of VL was found to be associated with the density of cattle or its ownership in India [12,13]. Serological evidences of anti-*L*. *donovani *antibodies in different domestic animals including cattle were reported in Sudan [14]. In a recent study in Nepal, *Leishmania *DNA was detected in several domestic animals including cattle from an endemic area [15]. However, to date, no study has been conducted in Bangladesh to investigate the role of any domestic animal in VL transmission.

This study was aimed to investigate the evidence of anti-leishmanial antibodies in blood of domestic cattle from VL-endemic villages of Mymensingh district in Bangladesh. Molecular diagnostic tests were also performed to detect circulating parasite DNA in blood through polymerase chain reaction (PCR) and loop-mediated isothermal amplification (LAMP).

## Methods

### Study area

The study was conducted in Trishal upazila (subdistrict) of Mymensingh district in Bangladesh. Trishal has a land area of 339 sq km, with a population of 3.7 million. The annual incidence of kala-azar in Trishal ranges from 21 to 26 per 10,000 people per year [16].

### Sample-size

Results of a previous study with domestic and wild animals in Sudan showed that 21.4% had seropositivity against anti-*L. donovani *antibodies in cows [14]. However, in the absence of a similar study in the Indian subcontinent, we assumed that cattle might show 10% of seropositivity in our study. Based on this assumption, we calculated that 138 cattle would be required for our study [precession 5% and 95% confidence interval (CI)]. Sample-size was calculated using Windows^® ^version of the Epi Info 3.2.2 software.

### Sample collection from cattle

Blood samples were collected from cattle during August-September 2008. At the beginning, past (within last 3 months) and active (treatment ongoing or awaiting for treatment) VL and post-kala-azar dermal leishmaniasis (PKDL) patients were identified from the records of Trishal Upazila Health Complex, Mymensingh. A research team, consisting of an experienced veterinary doctor or a veterinary assistant, visited the study houses, enumerated domestic cattle (*Bos indicus*), and collected five mL of blood from the jugular vein from randomly-selected one from each study house. After collection, three mL of blood was transferred to an EDTA containing tube for Buffy coat separation, and the remaining two mL was transferred to a sterile test tube for serum separation. Relevant information on age, sex, body condition score, etc. and any changes in behaviour within the past six months of the selected cattle were recorded. Their physical condition was scored in the scale of 1-5 (with 0.5 fractions between 2 scores) which represent worst to best physical condition on the basis of bony prominence and deposition of subcutaneous fat [17]. The blood samples were transported to the nearby field office for Buffy coat and serum separation. The processed samples were then preserved at -20°C before transferring these to the Parasitology Laboratory of ICDDR, B in Dhaka for further serological and molecular investigations.

### Ethical consideration

The study was approved by the Research Review Committee and the Animal Experimentation Ethics Committee of ICDDR, B. Consent was obtained from the owners of the cattle before the collection of blood sample.

### Laboratory investigation

#### Enzyme-linked immunosorbent assay

For the qualitative detection of antibodies (IgG, IgM, and IgA) against *L. donovani *in the serum samples, the RecombiLISA *Leishmania *Ab Test (CTK Biotech Inc., San Siego, USA) was performed. The RecombiLISA *Leishmania *Ab Test comprises two key components: (a) solid microwells pre-coated with recombinant *L. donovani *antigens and (b) liquid conjugates composed of recombinant *L. donovani *antigens conjugated with horse reddish peroxidase (HRP-*Leish *Ag conjugates). The test was performed by incubating serum and HRP-*Leish *Ag conjugate in wells of the plate. Antibodies (IgG, IgM, or IgA) to *L. donovani *in the specimens would react to the *L. donovani *antigens coated onto the plate well by making complex with the HRP-*Leish *Ag conjugates. Unbounded conjugates were then removed by washing. After adding substrate and stop solution, the change in color to yellow indicates Ab-positive, and then the absorbance was measured by spectrophotometer at 450 nm absorbance. Duplicate wells were run for each sample.

#### Interpretation of ELISA results

The ELISA results were interpreted according to the instructions of the manufacturer. Briefly, a cut-off value was obtained from the mean optical density (OD) value of the negative controls + 0.1. Then an OD ratio was calculated for each sample by dividing the mean OD value by the cut-off value. Sample with OD ratio of ≥ 1.00 was considered to be positive, and the OD ratio of < 1.00 was considered to be negative. Serum samples collected from four cattle from a non-endemic area were used as negative controls in each test. Repeated tests were performed with serum samples with a positive result along with an equal number of randomly-selected negative serum samples and negative controls to make a precise conclusion. However, no discrepancy was found in the repeat tests.

#### Direct agglutination test

The DAT was performed with the serum samples which were found to be positive in the ELISA test, along with a similar number of ELISA-negative samples. Commercially-available DAT antigen (Koninklijk Instituut voor de Tropen, Amsterdam, the Netherlands) was used following the protocol described by Harith *et al*. [18]. Duplicate wells were run for each of the serum dilution. Heat-inactive fetal bovine serum was used along with physiologic saline and DAT antigen as negative control [19]. An agglutination titer of 1:3200 was considered positive in this study [14].

#### PCR

DNA from Buffy coat was extracted using QIAamp^® ^DNA blood mini kit (QIAGEN, Düsseldorf, Germany). Nested PCR (Ln PCR) was performed to amplify the *ssu-rRNA *gene with some modifications of the protocol previously described by Cruz *et al*. [20]. The first PCR was specific for the order Kinetoplastida using the primer R221 and R332 with a product size of 603 base pair. The second PCR was specific for *Leishmania *genus using the diluted (1:50) first PCR product using the primers R223 and R333, with the product size of 358 base pair. Briefly, in the first reaction, 2 μL of the extracted DNA, 15 pmol of primers (Kinetoplastida-specific) R221 and R332 were used in 25 μL PCR mix containing 0.2 mM dNTP, 2 mM MgCl_2_, 5 mM KCl, 75 mM Tris-HCl (P^H ^9.0), 0.001% bovine serum albumin, 2.0 mM (NH_4_)_2_SO_4 _and 2.5 U of T*aq *polymerase. For the second reaction, 1 μL of a 1/50 dilution of the first PCR product was used as a template in the presence of 15 pmol of primer R223 and 15 pmol of primer R333 which are *Leishmania *genus-specific. Reaction conditions are the same as the first PCR. Amplification products were visualized on 1.5% agarose gel using a 100 bp DNA ladder after staining with ethidium bromide (0.1 mg/mL). DNA from in-house-cultured *L. donovani *was used as a positive control in the PCR assay.

#### Loop-mediated isothermal amplification

LAMP was performed followed the protocol described by Takagi *et al*. [21]. In brief, the LAMP reaction was performed in 25 μL of reaction mixture containing 40 pmol each of FIP and BIP primers, 5 pmol each of F3 and B3c primers, 1.4 mM each of deoxynucleoside triphosphate, 0.8 M betaine, 20 mM Tris-HCl, pH 8.8, 10 mM KCl, 10 mM (NH 4)_2 _SO_4_, 8 mM MgSO_4_, 0.1% TritonX-100, 8 units of *Bst *DNA polymerase large fragment (New England Biolabs, Ipswich, MA), and 2 μL of sample DNA. The mixture was incubated at 65°C for 50 minutes in a heat block. After incubation, the turbidity was examined visually.

#### Data analysis

Data were computed in the Microsoft Excel software. Frequencies and the association between different variables (chi-square test with Yate's correction and Fisher's exact test) were analyzed using the SPSS software (version 11.5). A p value of < 0.05 was considered significant.

## Results

Blood samples were collected from 138 cattle (50 males and 88 females). The median number of cattle per household was two, ranging from one to ten. Their mean age was 31.7 (range 8-144) months. Most (94.9%) were native breed. Fifty-three (38.4%) had the physical condition score of 2 (Table 1). Six cattle (4.3%) had visible lesions in their body, which were mostly myiasis (Table 1).

**Table 1:** Some characteristics of the study cattle along with ELISA results

Category		Frequency (%)	Positive (%)	
**Sex**				
Male	50 (36.2)			5 (3.6)
Female	88 (63.8)			8 (5.8)

**Age-group (months)**				
10 or less	4 (2.9)			0 (0)
11-20	42 (30.4)			5 (3.6)
21-30	40 (29.0)			1 (0.7)
31-40	23 (16.7)			1 (0.7)
41-50	11 (8.0)			2 (1.4)
51 plus	18 (13.0)			4 (2.9)

**Physical condition score**				
1	17 (12.3)			0 (0)
2	53 (38.4)			4 (2.9)
3	38 (27.5)			4 (2.9)
4	23(16.7)			4 (2.9)
5	7 (5.1)			1 (0.7)

**Lesions**				
None	132 (95.7)			12 (8.7)
Scar marks	3 (2.2)			0 (0)
Myiasis	2 (1.4)			0 (0)
Skin rash	1 (0.7)			0 (0)

**Total**	138 (100)			13 (9.4)

**Figure 1:**
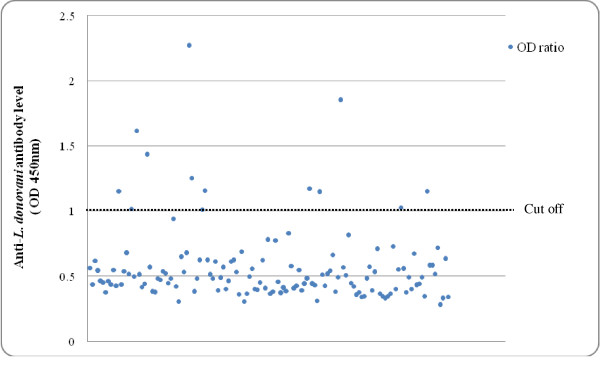
ELISA reading for the presence of anti *L. donovani *antibodies

Thirteen (9.4%) serum samples were found to be positive in ELISA (Table 1; Figure 1). The positive rate was the highest in the age group of 11-20 months (3.6%), followed by the age-group of 51+ months (Table 1). No statistical association was observed between seropositive cattle and their age-group (p = 0.279), sex (p = 0.1) or physical condition score (p = 0.413).

Due to limited resources, DAT was performed only with 13 positive samples and a similar number of negative samples. Only four (30.8%) of the 13 samples were positive in DAT (Table 2). None of the sample was positive in Ln PCR and LAMP.

**Table 2:** Profile of ELISA-positive cattle

ID	Age (months)	Sex	Colour	PCS*	Variety	Lesion	DAT** titer	DAT result
12	36	Female	Reddish	2	Native	Myiasis	1:1600	-ve
17	60	Female	Reddish	4	Native	No	1:800	-ve
19	72	Female	White	5	Native	No	1:3200	+ve
23	12	Female	White	3	Native	No	1:1600	-ve
39	18	Male	Reddish	3	Native	No	1:6400	+ve
40	18	Male	Brown	2	Red Shindhi	No	1:1600	-ve
44	15	Male	Reddish brown	2	Red Shindhi	No	1:200	-ve
45	14	Male	White	3	Native	No	1:3200	+ve
85	48	Female	Reddish	4	Native	No	1:1600	-ve
89	60	Female	Reddish and white	3	Native	No	1:400	-ve
97	30	Male	White	2	Native	No	1:3200	+ve
120	42	Female	Reddish	4	Native	No	1:800	-ve
130	60	Female	White	4	Native	No	1:1600	-ve

* Physical condition score; **direct agglutination tests

## Discussion

This is the first study in Bangladesh to investigate any domestic animal for VL infection. Our results showed that cattle produced antibodies which might be due to their exposure to the *Leishmania *parasite through infected sandfly bites but were unable to produce any infection. The absence of parasite DNA in molecular assays might be due to failure of the parasite to survive or propagate in the host.

Earlier in India, search for possible animal reservoir for VL mostly targeted to dogs based on direct observation of smears for amastigotes from peripheral blood, liver, spleen, and bone marrow [22-24]. Chakravarty *et al*. surveyed 64 cows along with dog, but could not find any amastigote in the smears [23].

At present, ELISA and DAT are two commonly-used serological methods for screening domestic and wild animals for the detection of anti-*Leishmania *antibodies. Due to insufficient information and lack of previous investigation in the Indian subcontinent; we adapted a cut-off titer of 1:3200 for DAT which was previously used in Sudan for screening anti-*L. donovani *antibodies in several domestic and wild species, including cows [14].

The presence of anti-*L. donovani *antibodies in ELISA and DAT of the cattle serum in our study supports the finding of a previous study in Sudan [14]. In Brazil, anti-*Leishmania *antibodies were found in domestic swine but were apparently resistant to *Leishmania *infection which may also occur in the case of cattle [25].

Antibody response in the cattle might also be due to the cross-reacting antibodies inferred by other infections which have been observed previously in human sera. For example anti-*Leishmania *antibodies were found cross-reactive with tuberculosis, toxoplasmosis, and malaria [26,27]. However, in this study, no such investigation for cross reactivity was done. The antibodies response in the few positive cattle in our study was quite weak which might be due to undiluted sera used in ELISA.

In a recent study in Nepal, *Leishmania *DNA was detected in domestic animals, such as goats, cows, and buffaloes, several months after the active transmission season [15]. We could not follow up our study cattle for detection of the parasite DNA in the following months due to limited resources which is a limitation of our study.

## Conclusions

Based on the findings of our study, it can be suggested that cattle are not a reservoir host for *L. donovani *despite its preference by *P. argentipes *as blood source. However, further studies need to be carried out to investigate the role of other domestic animals in VL epidemiology in this region.

## Competing interests

The authors declare that they have no competing interests.

## Authors' contributions

MSA, DG, MGMK, MFI, DM, MNI, and RH were responsible for the study design; MSA, DG, MGMK, MFI, and MNI were responsible for the collection of biological samples and clinical examination of animals; MSA, MGMK, MFI, MI, and RH were responsible for the execution of the laboratory work; MSA, DG, MGMK, DM, MI, and RH drafted the manuscript. All the authors critically revised the manuscript for intellectual content, and read and approved the final version.

## References

[B1] WHO Report of the Fifth Consultative Meeting on *Leishmania*/HIV Co infection 2007 Addis Ababa, Ethiopia: World Health Organization

[B2] WHO/TDR Research to support the elimination of visceral leishmaniasis Annual Report 2008 2009 World Health Organization 27

[B3] Rijal S Koirala S Van der Stuyft P Boelaert M The economic burden of visceral leishmaniasis for households in Nepal Trans R Soc Trop Med Hyg 2006 100 9 838 841 10.1016/j.trstmh.2005.09.017 16406035

[B4] Das S Boreham PF Bhattacharya NC Sen Gupta PC Prevalence and blood meal sources of *Phlebotomus argentipes *in West Bengal in 1972-73 Indian J Med Res 1976 64 9 1307 1313 1010623

[B5] Mukhopadhyay AK Chakravarty AK Bloodmeal preference of *Phlebotomus **argentipes *&*Ph. papatasi *of north Bihar, India Indian J Med Res 1987 86 475 480 3127337

[B6] Mukhopadhyay AK Kumar K Rahman SJ Host preference of phlebotomine sandflies in and around Delhi J Commun Dis 1987 19 4 408 411 3507449

[B7] Palit A Bhattacharya SK Kundu SN Host preference of *Phlebotomus argentipes *and *Phlebotomus papatasi *in different biotopes of West Bengal, India Int J Environ Health Res 2005 15 6 449 454 10.1080/09603120500392525 16506438

[B8] Pandya AP Bloodmeals of phlebotomine sandflies of Surat district (Gujarat state) India Indian J Med Res 1985 81 46 48 3988328

[B9] Bern C Courtenay O Alvar J Of cattle, sand flies and men: a systematic review of risk factor analyses for South Asian visceral leishmaniasis and implications for elimination PLoS Negl Trop Dis 2010 4 2 e599 10.1371/journal.pntd.0000599 20161727 PMC2817719

[B10] Bern C Hightower AW Chowdhury R Ali M Amann J Wagatsuma Y Haque R Kurkjian K Vaz LE Begum M Akter T Cetre-Sossah CB Ahluwalia IB Dotson E Secor WE Breiman RF Maguire JH Risk factors for kala-azar in Bangladesh Emerg Infect Dis 2005 11 5 655 662 15890115 10.3201/eid1105.040718 PMC3320384

[B11] Bern C Joshi AB Jha SN Das ML Hightower A Thakur GD Bista MB Factors associated with visceral leishmaniasis in Nepal: bed-net use is strongly protective Am J Trop Med Hyg 2000 63 3-4 184 188 11388512 10.4269/ajtmh.2000.63.184

[B12] Barnett PG Singh SP Bern C Hightower AW Sundar S Virgin soil: the spread of visceral leishmaniasis into Uttar Pradesh, India Am J Trop Med Hyg 2005 73 4 720 725 16222016

[B13] Saha S Ramachandran R Hutin YJ Gupte MD Visceral leishmaniasis is preventable in a highly endemic village in West Bengal, India Trans R Soc Trop Med Hyg 2009 103 7 737 742 10.1016/j.trstmh.2008.10.006 19036393

[B14] Mukhtar MM Sharief AH el Saffi SH Harith AE Higazzi TB Adam AM Abdalla HS Detection of antibodies to *Leishmania donovani *in animals in a kala-azar endemic region in eastern Sudan: a preliminary report Trans R Soc Trop Med Hyg 2000 94 1 33 36 10.1016/S0035-9203(00)90429-2 10748894

[B15] Bhattarai NR Van der Auwera G Rijal S Picado A Speybroeck N Khanal B De Doncker S Das ML Ostyn B Davies C Coosemans M Berkvens D Boelaert M Dujardin JC Domestic animals and epidemiology of visceral leishmaniasis, Nepal Emerg Infect Dis 2010 16 2 231 237 20113552 10.3201/eid1602.090623 PMC2958000

[B16] Bern C Chowdhury R The epidemiology of visceral leishmaniasis in Bangladesh: prospects for improved control Indian J Med Res 2006 123 3 275 288 16778310

[B17] Nicholson MJ Butterworth MH A guide to condition scoring of zebu cattle 1986 Agribookstore/Winrock

[B18] Harith AE Kolk AH Kager PA Leeuwenburg J Muigai R Kiugu S Laarman JJ A simple and economical direct agglutination test for serodiagnosis and sero-epidemiological studies of visceral leishmaniasis Trans R Soc Trop Med Hyg 1986 80 4 583 536 10.1016/0035-9203(86)90149-5 3101241

[B19] Islam MZ Itoh M Mirza R Ahmed I Ekram AR Sarder AH Shamsuzzaman SM Hashiguchi Y Kimura E Direct agglutination test with urine samples for the diagnosis of visceral leishmaniasis Am J Trop Med Hyg 2004 70 1 78 82 14971702

[B20] Cruz I Canavate C Rubio JM Morales MA Chicharro C Laguna F Jimenez-Mejias M Sirera G Videla S Alvar J A nested polymerase chain reaction (Ln-PCR) for diagnosing and monitoring *Leishmania infantum *infection in patients co-infected with human immunodeficiency virus Trans R Soc Trop Med Hyg 2002 96 Suppl 1 S185 189 12055836 10.1016/s0035-9203(02)90074-x

[B21] Takagi H Itoh M Islam MZ Razzaque A Ekram AR Hashighuchi Y Noiri E Kimura E Sensitive, specific, and rapid detection of *Leishmania donovani *DNA by loop-mediated isothermal amplification Am J Trop Med Hyg 2009 81 4 578 582 10.4269/ajtmh.2009.09-0145 19815869

[B22] Bhattacharya A Ghosh TN A search for *Leishmania *in vertebrates from kala-azar-affected areas of Bihar, India Trans R Soc Trop Med Hyg 1983 77 6 874 875 6665843 10.1016/0035-9203(83)90313-9

[B23] Chakravarty AK Sanyat RK Suri JC Zoonotic reservoir in Indian kala-azar J Commun Dis 1979 11 219 220

[B24] Zahar AR Studies Leishmaniasis, Vectors/Reservoirs and their control in the old world; Part IV. Asia and Pacific 1980 87 World Health Organization

[B25] Moraes-Silva E Antunes FR Rodrigues MS da Silva Juliao F Dias-Lima AG Lemos-de-Sousa V de Alcantara AC Reis EA Nakatani M Badaro R Reis MG Pontes-de-Carvalho L Franke CR Domestic swine in a visceral leishmaniasis endemic area produce antibodies against multiple *Leishmania infantum *antigens but apparently resist to *L. infantum *infection Acta Trop 2006 98 2 176 182 10.1016/j.actatropica.2006.04.002 16730628

[B26] Badaro R Eulalio MC Benson D Freire M Miranda JC Pedral-Sampaio D Burns JM David JR Johnson WD Reed SG Sensitivity and specificity of a recombinant *Leishmania chagasi *antigen in the serodiagnosis of visceral leishmaniasis Arch Inst Pasteur Tunis 1993 70 3-4 331 332 7802487

[B27] Burns JM Shreffler WG Benson DR Ghalib HW Badaro R Reed SG Molecular characterization of a kinesin-related antigen of *Leishmania chagasi *that detects specific antibody in African and American visceral leishmaniasis Proc Natl Acad Sci USA 1993 90 2 775 779 10.1073/pnas.90.2.775 8421715 PMC45748

